# Controlled Carbon Loss: Threshold-Dependent Overflow Metabolism in *Synechocystis* sp. PCC 6803

**DOI:** 10.3390/microorganisms13122767

**Published:** 2025-12-04

**Authors:** Janette T. Alford, Nathalie S. Becker, Johanna Rapp, Andreas Kulik, Janine Kaewbai-ngam, Tanakarn Monshupanee, Hannes Link, Karl Forchhammer

**Affiliations:** 1 Microbiology and Organismic Interactions, Interfaculty Institute of Microbiology and Infection Medicine, University of Tübingen, Auf der Morgenstelle 28, 72076 Tübingen, Germany; janette-theresa.alford@uni-tuebingen.de (J.T.A.); nathalie.becker@uni-tuebingen.de (N.S.B.); 2 Bacterial Metabolomics, Interfaculty Institute of Microbiology and Infection Medicine, University of Tübingen, Otfried-Müller-Straße 37, 72076 Tübingen, Germany; johanna.rapp@uni-tuebingen.de (J.R.); hannes.link@uni-tuebingen.de (H.L.); 3 Microbial Bioactive Compounds, Interfaculty Institute of Microbiology and Infection Medicine, University of Tübingen, Auf der Morgenstelle 28, 72076 Tübingen, Germany; andreas.kulik@uni-tuebingen.de; 4 Biochemistry, Faculty of Science, Chulalongkorn University, Bangkok 10330, Thailand; jkaewbaingam@gmail.com (J.K.-n.); tanakarn.m@chula.ac.th (T.M.)

**Keywords:** carbon metabolism, glycogen, overflow metabolism, *Synechocystis*

## Abstract

Cyanobacteria such as *Synechocystis* sp. PCC 6803 are promising chassis for sustainable bioproduction. During nitrogen starvation, *Synechocystis* redirects fixed carbon from biomass growth toward glycogen accumulation as a carbon and energy reserve. Inhibiting glycogen synthesis results in the excretion of excess carbon as organic acids, predominantly pyruvate and 2-oxoglutarate. Efficiently rerouting this carbon toward the formation of value-added products such as the plastic alternative polyhydroxybutyrate requires a deeper understanding of carbon partitioning and overflow metabolism. To investigate this, we quantified intra- and extracellular metabolites in *Synechocystis* wild-type and mutant strains with altered glycogen metabolism (Δ*pgm*, Δ*glgC*, Δ*glgA1*, Δ*glgA2*), nitrogen signaling (Δ*glnB*), and carbon allocation (Δ*pirC*), including the double mutant Δ*glgC*Δ*pirC*. Metabolites were analyzed after two days of nitrogen-replete or -depleted growth using enzymatic glycogen quantification and liquid chromatography-mass spectrometry. Excretion was primarily triggered by inhibition of glycogen synthesis but modulated by other changes in carbon flow, such as *pirC* deletion. Besides pyruvate and 2-oxoglutarate, small amounts of glutamate, succinate, and malate were excreted. Our findings suggest that, rather than a passive consequence of metabolite accumulation, excretion is a selective, threshold-dependent process that limits intracellular metabolite buildup, revealing an additional layer of metabolic control relevant to cyanobacterial bioengineering.

## 1. Introduction

Approximately 2.4 billion years ago, cyanobacteria initiated one of the most significant developments in Earth’s history. By evolving the capability to perform oxygenic photosynthesis, they were the driving force behind the change in the planet’s atmosphere, generating oxygen and thereby facilitating aerobic life and enabling the evolution of life as we know it [1]. Today, due to their ability to utilize light energy to sequester CO_2_, cyanobacteria could play a crucial role in shaping biotechnology as green, sustainable cell factories. As the only photosynthetic bacteria capable of oxygenic photosynthesis [2], cyanobacteria are promising platforms for sustainable bioproduction. They harness light energy to efficiently convert CO_2_ into value-added products such as acetone, ethanol, sucrose, succinate, or the plastic alternative polyhydroxybutyrate (PHB), releasing oxygen as the sole by-product [3]. However, improving production efficiency and reducing costs requires bioengineering [4]. A deeper understanding of cyanobacterial carbon flux is fundamental to optimizing these efforts.

One of the most commonly used host strains for pathway engineering studies of cyanobacteria is the model organism *Synechocystis* sp. PCC 6803 (*Synechocystis*) [3]. In non-diazotrophic cyanobacteria, such as *Synechocystis*, starvation of the essential nutrient nitrogen is a powerful driving force to switch metabolism from biomass growth to desired product formation [5,6,7]. To increase the yield of products such as PHB, lactate, or succinate, carbon needs to be redirected from the glycogen reserve into product synthesis [5,8,9]. As a non-diazotrophic cyanobacterium, *Synechocystis* is unable to fix atmospheric nitrogen and therefore requires a combined nitrogen source for growth. Upon encountering nitrogen-limiting conditions, *Synechocystis* initiates a survival program termed chlorosis, resulting in cells entering a long-lived dormant state [10]. During the initial phase of chlorosis, *Synechocystis* degrades its light-harvesting complexes, the phycobilisomes, resulting in a color change of the cells from green to yellow and strongly reduced photosynthetic activity [11,12,13,14]. Concomitantly, the cells accumulate glycogen reserves as carbon and energy storage to survive prolonged nitrogen starvation and support a highly orchestrated resuscitation process once a nitrogen source becomes available again [10,15].

Blocking glycogen as an energy and carbon sink in *Synechocystis* is most commonly achieved by deleting the *glgC* gene, but can also be accomplished by deleting *pgm* [16] or by simultaneous deletion of *glgA1* and *glgA2* [17]. The phosphoglucomutase (PGM) encoded by *pgm* connects glycogen metabolism to the central carbon metabolism by catalyzing the interconversion of glucose-6-phosphate and glucose-1-phosphate. While the glucose-1-phosphate adenylyltransferase (GlgC) encoded by *glgC* catalyzes the conversion of glucose-1-phosphate to ADP-glucose, the first committed, irreversible step of glycogen synthesis, the two glycogen synthase isoenzymes GlgA1 and GlgA2 assemble the resulting ADP-glucose monomers into glycogen polymers.

Obstructing carbon flow into the glycogen sink results in the redirection of photosynthetically fixed carbon into alternative carbon sinks during nutrient starvation. A *Synechocystis* strain lacking *glgC* displays growth comparable to the wild type (WT) under continuous illumination and nitrogen-replete conditions [17,18]. However, upon encountering nitrogen starvation, unlike the WT, Δ*glgC* is unable to accumulate glycogen and therefore is impaired in executing the chlorosis program [17]. Furthermore, under nitrogen-starved conditions, deletion of *glgC* results in the excretion of photosynthetically fixed carbon as organic acids, mainly 2-oxoglutarate (2-OG) and pyruvate, rather than its storage as glycogen. This overflow metabolism suggests that if glycogen synthesis is impaired during nitrogen starvation, the cells are overloaded with carbon influx and excrete products in response [18].

Overflow metabolism has been extensively studied in heterotrophic bacteria and higher organisms, where it serves as an energy-spilling mechanism when catabolism exceeds anabolism [19]. It typically occurs under excess glucose conditions and is characterized by excretion of fermentation products like ethanol, acetate, lactate, as well as intermediates from glycolysis, the pentose-phosphate pathway, the TCA cycle, and free amino acids. Accumulation of these metabolites is particularly pronounced under nitrogen-limited conditions [20]. For the photoautotrophic *Synechocystis*, metabolic overflow into the medium has been predominantly observed as a result of impaired glycogen metabolism during nitrogen depletion [17], but could also be triggered by cultivation under high-light in nitrogen-repleted conditions [21]. These findings led to the hypothesis that metabolite overflow in *Synechocystis* functions as a mechanism for energy or redox balancing as well as an alternative carbon sink [17,21].

Here, we aimed to gain a deeper insight into the carbon flow and metabolite excretion of *Synechocystis* by extensively analyzing the changes within intracellular and extracellular metabolites upon nitrogen starvation across various *Synechocystis* carbon metabolism mutants. Particular emphasis was placed on investigating a regulatory mutation of the phosphoglycerate mutase (PGAM) reaction. The PGAM reaction directs the first product of CO_2_ fixation, 3-phosphoglycerate, towards lower glycolysis to replenish numerous anabolic pathways. During nitrogen starvation, PGAM activity is inhibited by the small protein PirC (P_II_-interacting regulator of carbon metabolism), which is under the control of the carbon/nitrogen/energy balance-sensing signaling protein P_II_ [22,23]. A *pirC* knock-out mutant (Δ*pirC*) lacks this inhibition, maintaining high PGAM activity and increased carbon flow into lower glycolysis during nitrogen-depletion, resulting in reduced glycogen accumulation and overproduction of the biopolymer PHB during chlorosis [22]. In addition to the Δ*pirC* mutant, we analyzed the single knockout strains Δ*glgA1*, Δ*glgA2*, Δ*glgC*, Δ*pgm*, and Δ*glnB*, as well as a Δ*glgC*Δ*pirC* double-knockout strain. Deletion of *glgA1*, *glgA2*, *glgC*, or *pgm* blocks the glycogen sink to different extents, thereby disrupting the main carbon sink. Since *glgC* deletion completely inhibits glycogen synthesis, concomitant deletion of *glgC* and *pirC* could potentially further amplify carbon flow into lower glycolysis and downstream metabolites. The Δ*glnB* strain, lacking P_II_, can no longer sense nitrogen stress or properly coordinate the C/N response and can therefore no longer orchestrate the appropriate response [24].

Using a combination of enzymatic glycogen quantification, high-performance liquid chromatography-mass spectrometry (HPLC-MS), and targeted liquid chromatography-tandem mass spectrometry (LC-MS/MS), we investigated the changes in intracellular and extracellular metabolites between vegetative and nitrogen-depleted carbon flux mutants and WT strains, to reveal which other disruptions of carbon flow result in metabolite overflow and potentially identify the metabolic changes triggering this response.

## 2. Materials and Methods

### 2.1. Cultivation of Synechocystis sp. PCC 6803

All strains used in this study are listed in Table S1. For vegetative cultivation, the strains were inoculated to an optical density (OD) of 0.15 at 750 nm and cultivated for two days in BG_11_ medium [25] supplemented with 5 mM NaHCO_3_. To induce nitrogen depletion, the strains were transferred from BG_11_ medium into BG_0_ medium, which contains the same components as BG_11_ medium, except for NaNO_3_. The transfer of strains from BG_11_ into BG_0_ medium was performed via a previously described two-step wash with BG_0_ [26], and strains were inoculated to an OD_750_ of 0.4 and cultivated for two days in BG_0_ medium. Vegetative and nitrogen-depleted cultures were prepared from the same exponentially growing precultures and adjusted to different initial ODs to ensure comparable cell densities at the time of sampling. Appropriate concentrations of antibiotics were added to the pre-cultures of all mutants to ensure strain integrity. All cultivations were conducted in a volume of 50 mL, in flasks shaken at 125 rpm and 28 °C, under continuous illumination (40–50 μmol photons m^−2^ s^−1^).

### 2.2. Glycogen Quantification

Glycogen content was quantified via a method combining the procedure established by Vidal et al. [27] with minor modifications based on the protocol described by Ortega-Martínez et al. [28]. For each sample, 0.5 mL of culture was harvested by centrifugation at 16,000× *g* for 5 min. After discarding the supernatant, the cell pellet was dried at 60 °C overnight in a heating cabinet and frozen at −20 °C for long-term storage. For cell lysis, the dry pellet was resuspended in 50 µL H_2_O, then 200 µL 30% (*w*/*v*) KOH was added, and the cells were incubated at 90 °C for 30 min. To neutralize the samples, 80 µL glacial acetic acid was added. After the samples cooled to room temperature, 670 µL of enzyme mix containing amyloglucosidase (AMG) (10115), glucose oxidase (GO) (G7141), peroxidase (PX) (P6782), and *o*-dianisidine dihydrochloride (ODD) (D3252) (Sigma Aldrich, St. Louis, MO, USA) in sodium acetate buffer (2.5 mM, pH 5.2) were added to each sample to a final concentration of 10 U AMG, 25 U GO, 5 U PX, and 0.2 mg/mL ODD and a final volume of 1 mL. To generate a calibration curve, a glycogen dilution series (0–200 µg/mL) was prepared, and 670 µL of enzyme mix was added to 330 µL of each dilution. The samples and the glycogen dilution series were incubated for 30 min at 37 °C with the enzyme mix, then 250 µL 12 M H_2_SO_4_ was added to a final concentration of 4.8 N to stop the reaction. Finally, the samples were centrifuged for 5 min at 16,000× *g*, and 200 µL of the resulting supernatants were used to determine the absorption at 540 nm in a 96-well plate using a TECAN Spark Multiplate reader (Tecan Group AG, Männedorf, Switzerland). Glycogen concentrations were calculated utilizing the calibration curve resulting from the measurement of the glycogen dilution series.

### 2.3. Quantification of Intracellular Metabolites

Cells were sampled after two days of vegetative or two days of nitrogen-depleted growth. To determine intracellular metabolite concentrations from comparable cell amounts, 10 mL of vegetatively grown cultures and 15 mL of nitrogen-depleted cultures were collected and processed as previously described by Doello et al. [29]. Metabolomic analysis was performed via targeted liquid chromatography-tandem mass spectrometry (LC-MS/MS) (Agilent TQ6495, Santa Clara, CA, USA) using an isotope ratio approach as described in Guder et al. [30]. The resulting C12/C13 ratios were used to calculate log_2_ fold changes in metabolite levels relative to the respective WT under vegetative conditions and visualized using GraphPad Prism 10.

### 2.4. Quantification of Extracellular Metabolites

To quantify extracellular metabolites, cultures were sampled in parallel with the sampling for intracellular metabolites after two days of vegetative or two days of nitrogen-depleted growth. For each sample, 2 mL of culture were centrifuged for 5 min at 10,000× *g* and 4 °C. Afterward, 400 µL of each supernatant was transferred into a vial and stored at 4 °C until the samples were measured on the same day. The samples were analyzed by high-performance liquid chromatography-mass spectrometry (HPLC-MS) using a high-performance liquid chromatography System Agilent 1200 series coupled with an Ultra Trap system XCT 6330 (Agilent Technologies, Waldbronn, Germany). Separation of the metabolites was achieved using a Luna Phenyl Hexyl 5 µm, 250 × 2 mm ID column (Phenomenex, Aschaffenburg, Germany) with the following HPLC parameters: column temperature 40 °C; mobile Phase: A = 0.1% formic acid in water, B = 0.06% formic acid in acetonitrile; gradient: t0 = t6 = 0% B, t11 = t18 = 100% B, post time 15 min 100% A; flowrate: 400 µL/min; and injection volume: 2.5 µL. The separated metabolites were measured in Electrospray Ionization negative ion ultra scan mode with a capillary voltage of 3.5 kV, and a mass scan of 50 to 300 *m*/*z*. Metabolite concentrations were calculated from a calibration curve generated by measuring standards with concentrations ranging from 0.01 mM to 0.1 mM of the metabolites of interest (malate, succinate, pyruvate, 2-OG, and glutamic acid) in the same way as the supernatant samples. Results were analyzed with the software 6300 series trap control version 6.1 Bruker Daltonics (Agilent, Santa Clara, CA, USA) and plotted with GraphPad Prism 10.

## 3. Results

### 3.1. Extracellular Metabolite Levels Do Not Simply Mirror Intracellular Metabolite Levels

To investigate overflow excretion of metabolites within *Synechocystis*, the accumulation of pyruvate, succinate, 2-OG, malate, and glutamate within the medium of *Synechocystis* Δ*glgA1*, Δ*glgA2*, Δ*glgC*, Δ*pgm*, Δ*pirC*, Δ*glnB*, and Δ*glgC*Δ*pirC* strains was measured during vegetative and nitrogen-depleted growth. In addition, the corresponding intracellular dynamics of these metabolites were determined to investigate the relation between intracellular accumulation and excretion of metabolites. As controls, we analyzed metabolite levels in two *Synechocystis* WT strains. Most mutants were generated in a glucose-tolerant (GT) WT (WT-GT) background. However, since the Δ*glnB*, Δ*glgC*, and Δ*glgC*Δ*pirC* deletions could not be obtained in this background, a glucose-sensitive (GS) WT (WT-GS) was included as an additional control. In addition, because intracellular pyruvate levels could not be captured by our analytical method, the levels of the neighboring metabolites phosphoenolpyruvate (PEP) and acetyl-CoA were used as an approximation for changes in the intracellular pyruvate pool. To compare the metabolic state of the selected strains during vegetative growth and nitrogen starvation, cells from an exponentially growing preculture were used to inoculate nitrogen-deficient and nitrogen-replete cultures, which were sampled after two days of vegetative growth or two days of nitrogen starvation, as cells should have completed their adaptation to nitrogen depletion within 48 h [13]. Importantly, even glycogen-free strains remain viable after two days of nitrogen starvation, despite their inability to properly adapt, with viability only decreasing after prolonged starvation [16,17]. Since overflow metabolism in cyanobacteria is triggered by disruption of the glycogen sink [17,28,31], we also measured the amount of glycogen accumulation in the analyzed strains under the different conditions.

**Figure 1:**
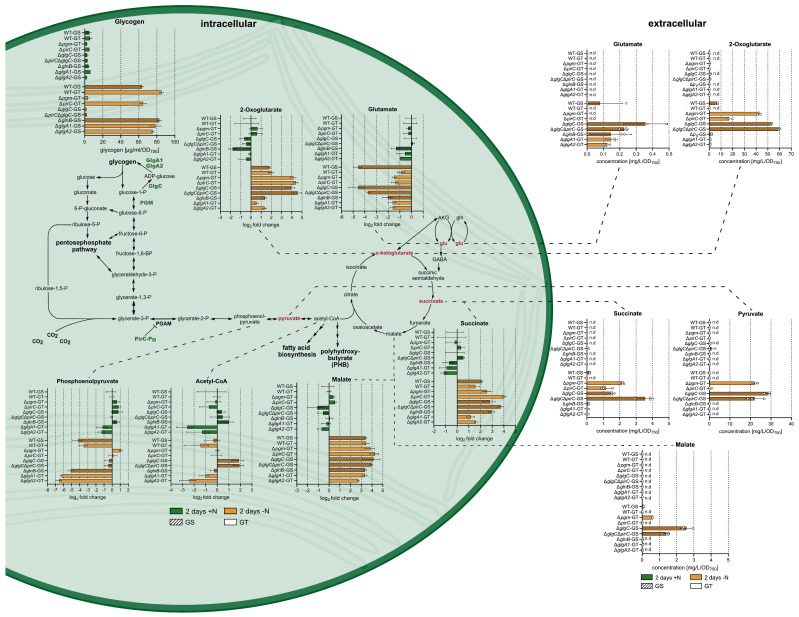
Comparison of intracellular changes of selected metabolite levels with the amounts of excreted metabolites. The intracellular values represent log_2_-fold changes in metabolites for WT-GS, WT-GT, Δ*pgm*-GT, Δ*pirC*-GT, Δ*glgC*-GS, Δ*glgC*Δ*pirC*-GS, Δ*glnB*-GS, Δ*glgA1*-GT, and Δ*glgA2*-GT after 2 days of vegetative growth (green) or 2 days of nitrogen starvation (orange), relative to the metabolite levels of the corresponding WT during vegetative growth. Each bar represents the mean log_2_-fold change of a triplicate, including the negative and positive standard deviation (SD). The extracellular values represent the mean metabolite concentration normalized to OD_750_ (Table S2) and calculated from triplicates including the SD; the individual values of the biological replicates are depicted as dots.

Quantification of glycogen confirmed that deletion of either *pgm* or *glgC* results in loss of glycogen accumulation during chlorosis, as both strains exhibited only marginal values close to the detection limit of the assay [27] (Figure 1). Simultaneously, deletion of *pirC* reduced the amount of accumulated glycogen moderately, in agreement with previous publications [16,17,22].

Our results also show that for all strains, metabolite excretion in noteworthy amounts only occurred during nitrogen starvation (Figure 1). As previously reported, during nitrogen starvation, considerable amounts of pyruvate and 2-OG were excreted by mutants unable to accumulate glycogen due to the deletion of *pgm* or *glgC* [17,28]. Specifically, Δ*pgm* released 22 mg/L/OD_750_ pyruvate and 42 mg/L/OD_750_ 2-OG, Δ*glgC* excreted 28 mg/L/OD_750_ and 53 mg/L/OD_750_, and Δ*glgC*Δ*pirC* excreted 22 mg/L/OD_750_ and 60 mg/L/OD_750_, respectively. Moreover, our results show that these strains also released small amounts of succinate and malate during nitrogen starvation, which, to our knowledge, has not been reported for *Synechocystis* before. In detail, Δ*glgC*Δ*pirC* released the highest amount of succinate (3.5 mg/L/OD_750_), followed by Δ*pgm* (2.1 mg/L/OD_750_) and Δ*glgC* (1.6 mg/L/OD_750_). For malate, Δ*glgC* showed the highest excretion (2.6 mg/L/OD_750_), whereas Δ*glgC*Δ*pirC* (1.4 mg/L/OD_750_) and Δ*pgm* (0.6 mg/L/OD_750_) released lower amounts. Interestingly, the Δ*pirC* strain also exhibited a distinct pattern of metabolite excretion. Despite retaining the ability to synthesize glycogen, albeit to lower levels than the corresponding WT, the Δ*pirC* strain excreted 17 mg/L/OD_750_ 2-OG and 1.2 mg/L/OD_750_ succinate, but neither pyruvate nor glutamate. The concurrent deletion of *glgC* and *pirC* resulted in an additive effect for succinate and 2-OG excretion, while extracellular pyruvate concentrations were comparable for the double-mutant and Δ*glgC*.

Remarkably, intracellular and extracellular metabolite dynamics did not always mirror each other. 2-OG was mainly excreted by the strains strongly accumulating 2-OG intracellularly, namely Δ*pgm*, Δ*pirC*, Δ*glgC*, and Δ*glgC*Δ*pirC*, and all strains excreting pyruvate had higher intracellular PEP and acetyl-CoA levels compared to the WT (Figure 1). However, not all strains with increased intracellular PEP and acetyl-CoA levels excreted pyruvate into the medium. This could be observed for the Δ*glgA1*, Δ*glnB*, and Δ*pirC* strains. Furthermore, succinate accumulated intracellularly in a similar way within the Δ*pirC* and the Δ*glgC*Δ*pirC* strain, but the latter excreted approximately three times more succinate into the medium. Malate was only excreted by the nitrogen-starved Δ*pgm*, Δ*glgC*, and Δ*glgC*Δ*pirC* strains, despite similar intracellular malate levels across all strains.

Intriguingly, glutamate was excreted even though intracellular levels declined under nitrogen starvation (Figure 1). Small amounts were excreted by one WT-GS replicate and by the corresponding Δ*glnB* mutant. The Δ*glgC* strain excreted the highest amount of glutamate, even though its intracellular glutamate levels dropped as strongly as in the cognate WT-GS. The Δ*glgC*Δ*pirC* strain showed a slightly smaller decrease in intracellular glutamate but nevertheless excreted similar amounts to the Δ*glgC* strain. Among the glucose-tolerant strains, intracellular glutamate decreased less than in the GS-background during nitrogen depletion. However, the Δ*glgA1* and Δ*glgA2* mutants excreted small amounts of glutamate, whereas Δ*pgm* and Δ*pirC* did not excrete any.

Altogether, these results show that metabolites were not simply excreted when they accumulated intracellularly, and metabolite excretion was not exclusively triggered by the blocking of the glycogen sink. Interestingly, metabolite excretion also did not categorically result in intracellular balancing of metabolite levels. Most strains that excreted metabolites did not exhibit intracellular metabolite levels comparable to those within the WT. Thus, metabolite excretion did not result in restoring intracellular metabolite levels to WT metabolite levels.

### 3.2. Intracellular Central Carbon and Nitrogen Metabolism

To further investigate which underlying carbon fluxes contribute to metabolite excretion, we analyzed the intracellular levels of 124 metabolites across all strains under both vegetative (Figure S1) and nitrogen-starved conditions (Figure S2). We focused on metabolites from central carbon and nitrogen metabolism captured by our measurement method, specifically comparing intermediates of glycolysis starting from glycogen, the TCA cycle, and the glutamine synthetase/glutamate synthase cycle (GS/GOGAT) during nitrogen starvation to the respective WT metabolite levels during vegetative growth.

**Figure 2:**
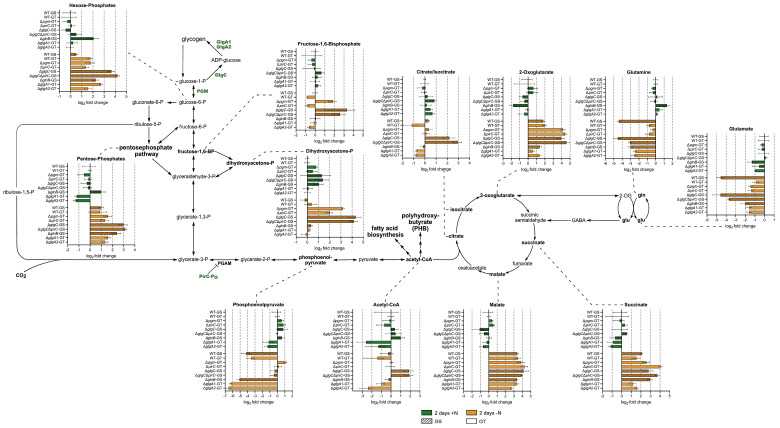
Carbon and nitrogen metabolism. Log_2_-fold changes in metabolites for WT-GS, WT-GT, Δ*pgm*-GT, Δ*pirC*-GT, Δ*glgC*-GS, Δ*glgC*Δ*pirC*-GS, Δ*glnB*-GS, Δ*glgA1*-GT, and Δ*glgA2*-GT after 2 days of vegetative growth (green) or 2 days of nitrogen starvation (orange), normalized to the metabolite levels of the corresponding WT during vegetative growth. Each bar represents the mean log_2_-fold change of a triplicate, including the negative and positive SD.

The results show that nitrogen depletion resulted in a buildup of hexose-phosphates and pentose-phosphates within all strains compared to their respective WT under vegetative growth conditions (Figure 2).

Interestingly, sugar-phosphate accumulation was more pronounced within strains lacking *glgC* as well as in the Δ*glnB* strain. Notably, Δ*glnB* exhibited higher intracellular levels of hexose-phosphates and pentose-phosphates than its cognate WT even during vegetative growth.

All strains displaying pronounced overflow metabolism, namely Δ*pgm*, Δ*glgC*, Δ*pirC*, and Δ*glgC*Δ*pirC*, exhibited higher intracellular dihydroxyacetone-phosphate (DHAP) levels than their respective WT during nitrogen starvation (Figure 2). Similarly, fructose-1,6-bisphosphate accumulated within all these strains, except Δ*pirC*, during nitrogen depletion.

In WT cells, PEP levels strongly decreased upon N-starvation (Figure 2). The same was observed for the Δ*glnB* and the Δ*glgA* mutants. Interestingly, those strains that export 2-OG (Δ*pgm*, Δ*pirC*, Δ*glgC*, and Δ*glgC*Δ*pirC*) maintained similar intracellular PEP levels during nitrogen depletion as during vegetative cultivation, or in the case of Δ*pgm*, even slightly increased PEP levels during nitrogen depletion.

Figure 2 also shows that all strains, including the WT strains, accumulated TCA cycle intermediates upon nitrogen starvation. However, while both WT accumulated predominantly malate, succinate, and 2-OG, but no or only slight amounts of citrate/isocitrate, especially Δ*glgC* and Δ*glgC*Δ*pirC* accumulated increased intracellular citrate/isocitrate levels during nitrogen depletion. The four strains excreting the most metabolites, Δ*pgm*, Δ*glgC*, Δ*pirC*, and Δ*glgC*Δ*pirC*, also displayed increased intracellular 2-OG levels during nitrogen-depleted growth, while the Δ*glgA1* and Δ*glgA2* strains had noticeably lower intracellular 2-OG levels during vegetative growth and nitrogen-depleted growth compared to the respective WT. During vegetative growth, lower intracellular 2-OG levels could also be observed for the Δ*glnB* strain.

While intracellular malate levels were comparable within all strains during nitrogen-limited cultivation, the accumulation of succinate was more pronounced in the Δ*glnB*, Δ*pirC*, and Δ*glgC*Δ*pirC*.

Within the main nitrogen assimilation pathway, the GS/GOGAT, both WT variants decreased their intracellular glutamine and glutamate levels during nitrogen starvation. However, there was a striking difference in the extent of this decrease between the WT-GT and WT-GS. While the WT-GS strongly decreased both metabolites during chlorosis, there was only a slight decrease in glutamine and glutamate levels within the WT-GT.

Within most mutant strains, glutamate dynamics upon nitrogen depletion were similar to those of their cognate WT. Noticeable exceptions were the Δ*glgC*Δ*pirC* and especially the Δ*glnB* strain, which decreased their intracellular glutamate level during nitrogen depletion not as strongly as the corresponding WT. This could also be observed for the glutamine dynamics within those strains. In contrast, the glutamine levels within the Δ*glgA* mutants decreased stronger than in their respective WT, while the glutamine dynamics of Δ*pgm*, Δ*pirC*, and Δ*glgC* during nitrogen depletion were comparable to those of their respective WT.

Collectively, these results suggest that metabolite excretion correlates with intracellular accumulation of key intermediates from glycolysis and the TCA cycle under nitrogen-starved conditions.

### 3.3. Intracellular Energy and Redox Balance

To determine whether our data supports the theory that overflow metabolism in *Synechocystis* is a mechanism for maintaining energy and redox balance [17,21,32], we examined the intracellular dynamics of AMP/ADP/ATP, NAD/NADH, NADP/NADPH, and reduced/oxidized glutathione in the various carbon metabolism mutants.

During nitrogen starvation, ATP, ADP, and AMP levels decreased in both WT strains (Table 1).

In contrast, all carbon metabolism mutants, except Δ*pirC*, showed a considerable increase in their intracellular AMP levels upon nitrogen shift (Table 1). This increase was strongest for the Δ*pgm* strain, which increased its AMP levels sixfold upon nitrogen starvation. Interestingly, all carbon metabolism mutants maintained similar or merely slightly altered ADP levels to those during vegetative growth under nitrogen-depleted conditions. These AMP and ADP dynamics were also reflected in the adenylate energy charge, calculated as (ATP + 0.5 ADP)/(ATP + ADP + AMP), of the various strains (Figure S3). Except for Δ*pirC*, the energy charge decreased in all strains during nitrogen starvation (Figure S3). Interestingly, the energy charge decrease was significantly more pronounced than in the respective WT for Δ*pgm* and Δ*glgA2*.

**Figure 3:**
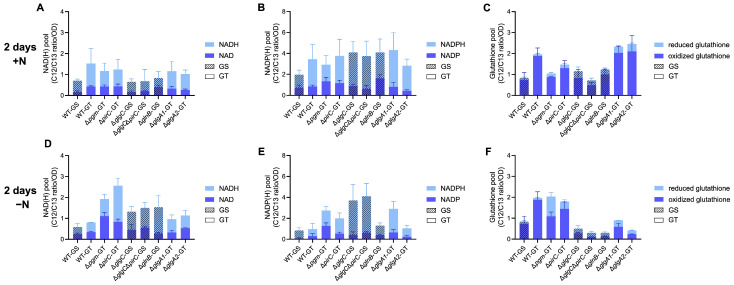
NAD(H) (**A**,**D**), NADP(H) (**B**,**E**), and reduced/oxidized glutathione pool (**C**,**F**) within WT-GS, WT-GT, Δ*pgm*-GT, Δ*pirC*-GT, Δ*glgC*-GS, Δ*glgC*Δ*pirC*-GS, Δ*glnB*-GS, Δ*glgA1*-GT, and Δ*glgA2*-GT during vegetative (**A**–**C**) and nitrogen-depleted growth (**D**–**F**). The bars represent the total amount of NAD(H), NADP(H), and glutathione, composed of the stacked values of the respective metabolite in their reduced (light blue) and oxidized (dark blue) form. Metabolite levels (C12/C13 ratios) were quantified via LC-MS/MS and normalized to OD_750_ at the time of sampling (Table S2). Each bar represents the mean of a triplicate, including the SD.

Investigation of the total NAD(H) pool revealed that total NAD(H) levels slightly decreased in both WT backgrounds upon nitrogen starvation. In contrast, Δ*pgm*, Δ*pirC*, Δ*glgC*, Δ*glgC*Δ*pirC*, and Δ*glnB* maintained high NAD(H) levels or even increased them (Figure 3A,D).

For the individual metabolites, NADH decreased slightly in both WT, while NAD remained mostly stable, whereas Δ*pgm*, Δ*pirC*, Δ*glgC*Δ*pirC*, and Δ*glgA2* exhibited increased NAD under nitrogen starvation. NADH levels were stable in Δ*pgm* and Δ*glgA2*, slightly increased in Δ*glgC*Δ*pirC* and Δ*glnB*, and noticeably increased in Δ*pirC*. Similarly, the total NADP(H) pool was comparable across strains during vegetative growth, except for a slightly elevated NADP level in Δ*glnB* (Figure 3B,E). Upon nitrogen depletion, total NADP(H) decreased in the WT strains, as well as in Δ*pirC*, Δ*glnB*, and Δ*glgA2*. In contrast, Δ*pgm*, Δ*glgC*, Δ*glgC*Δ*pirC*, and Δ*glgA1* maintained NADP(H) levels similar to those during vegetative growth. Simultaneously, the individual NADP and NADPH levels decreased in both WT and Δ*glgA2*, but remained stable in Δ*pgm*, Δ*glgC*, Δ*glgC*Δ*pirC*, Δ*pirC*, and Δ*glgA1*. Although NAD(H) and NADP(H) pools as well as individual cofactor levels varied across strains, the NAD/NADH and NADP/NADPH ratios were generally stable, with significant changes observed only for nitrogen-starved Δ*pgm* and Δ*glnB* (Figure S4). This suggests that most strains maintain their NAD(H)/NADP(H) redox balance despite altered cofactor pools.

We also investigated the redox state of glutathione within the different strains, as glutathione is a key molecule in the antioxidative defense system in cyanobacteria. It helps maintain protein cysteine residues in their reduced state and detoxifies reactive oxygen species, thereby protecting cells from oxidative damage. Glutathione is synthesized from glutamate, cysteine, and glycine, linking its function to glutamate metabolism [33,34,35].

In both WT strains and in Δ*pirC*, the total glutathione pool as well as its composition remained largely unchanged between vegetative growth and nitrogen starvation (Figure 3C,F). In contrast, Δ*glgC*, Δ*glgC*Δ*pirC*, Δ*glnB*, Δ*glgA1*, and Δ*glgA2* showed a decrease in total glutathione levels, whereas Δ*pgm* exhibited an increase in total glutathione. The drop of the glutathione pool in Δ*glnB*, Δ*glgA1*, and Δ*glgA2* was mainly driven by a strong decrease in oxidized glutathione, while in Δ*glgC* and Δ*glgC*Δ*pirC*, only a slight decrease in the oxidized fraction was observed. The increase in the glutathione pool in Δ*pgm*, by comparison, resulted from the accumulation of reduced glutathione under nitrogen starvation. So, in contrast to the largely stable NAD(H) and NADP(H) ratios, analysis of the glutathione redox balance indicates a strain-specific imbalance, with Δ*glnB*, Δ*glgA1*, and Δ*glgA2* displaying a more pronounced decrease in oxidized glutathione, while Δ*pgm* accumulated reduced glutathione.

Overall, these results show that most strains largely preserve energy and redox homeostasis during nitrogen depletion, whereas Δ*glnB*, Δ*glgA1*, and Δ*glgA2* show moderate imbalances, and Δ*pgm* displays the most pronounced disruption, combining reduced energy charge from AMP accumulation with a distinct shift toward reduced glutathione.

### 3.4. Metabolite Excretion Represents a Major Alternative Carbon Sink

Gründel et al. [17] previously proposed that overflow excretion provides an alternative carbon sink in the absence of glycogen storage. To assess how fixed carbon was partitioned between glycogen storage and metabolite excretion within the investigated strains, we converted measured glycogen amounts into intracellular glucose equivalents (Equation (S1)) and, analogously, calculated the intracellular metabolite concentrations removed from the cell via excretion from the extracellularly detected amounts of metabolites (Equation (S2)).

**Figure 4:**
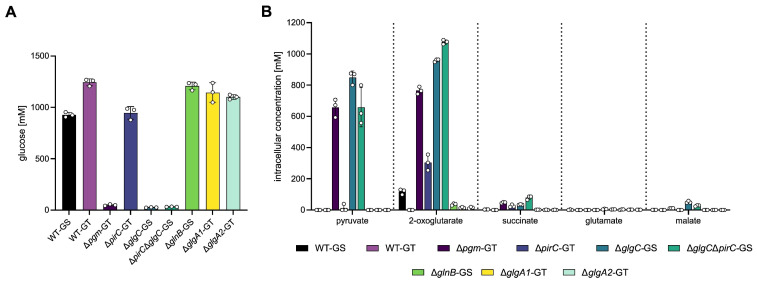
Conversion of measured glycogen amounts into intracellular glucose equivalents stored in form of glycogen per cell (**A**) and corresponding intracellular metabolite concentrations removed from each cell via excretion calculated from the extracellularly detected amounts of pyruvate, 2-OG, succinate, glutamate, and malate (**B**) for WT-GS (black), WT-GT (light purple), Δ*pgm*-GT (dark purple), Δ*pirC*-GT (blue), Δ*glgC*-GS (teal), Δ*glgC*Δ*pirC*-GS (green), Δ*glnB*-GS (bright green), Δ*glgA1*-GT (yellow), and Δ*glgA2*-GT (turquoise). Each bar represents the mean of a triplicate, including the SD. The individual values for each biological replicate are depicted as dots.

As shown in Figure 4A, WT-GS, WT-GT, Δ*pirC*, Δ*glnB*, Δ*glgA1*, and Δ*glgA2* accumulated glycogen corresponding to 0.93–1.25 M intracellular glucose under nitrogen starvation. While cells of the Δ*pgm* mutant failed to accumulate noteworthy amounts of glycogen, they instead excreted organic acids corresponding to an intercellular equivalent of ca. 657 mM pyruvate, 765 mM 2-OG, 48 mM succinate, and 12 mM malate (Figure 4B). Similarly, Δ*glgC* excreted an equivalent of ca. 848 mM pyruvate, 958 mM 2-OG, 35 mM succinate, and 51 mM malate, while Δ*glgC*Δ*pirC* released an equivalent of ca. 658 mM pyruvate, 1076 mM 2-OG, 79 mM succinate, and 28 mM malate.

Considering the carbon content of the excreted metabolites, Δ*pgm*, Δ*glgC*, and Δ*glgC*Δ*pirC* excreted approximately 1–1.3 M glucose equivalents, representing ca. 81%, 138%, and 140%, respectively, of the carbon that would have been stored as glycogen by the respective WT under the same conditions. This indicates that the fixed carbon normally directed into glycogen storage is redirected into overflow excretion when glycogen synthesis is impaired. Notably, among the three mutants, Δ*pgm* excreted the least carbon despite its equal inability to store glycogen, and deletion of *pirC* in the Δ*glgC* background did not lead to a substantial increase in carbon excretion.

## 4. Discussion

It has previously been shown that disrupting the glycogen sink within *Synechocystis* by deleting Δ*glgC* results in excretion of 2-OG and pyruvate, and that drastically reducing glycogen accumulation by deleting Δ*pgm* has a similar effect [17,28]. Our data not only confirmed this but also revealed that overflow metabolism within these strains is not limited to the excretion of 2-OG and pyruvate, as small amounts of malate, succinate, and, in the case of Δ*glgC*, also glutamate were additionally excreted. Nevertheless, pyruvate and 2-OG remain the most prominently excreted metabolites (Figure 1). This aligns with findings by Kato et al. [31], who reported that *Synechococcus elongatus* PCC 7942 excretes several metabolites in addition to 2-OG and pyruvate when glycogen synthesis is blocked during nitrogen starvation. Additionally, Kato et al. [31] showed that, in the presence of a nitrogen source, a *glgC*-deficient mutant of *Synechococcus elongatus* PCC 7942 primarily excreted glutamate as an alternative carbon sink to glycogen. Here, we show that the Δ*glgC* mutant in *Synechocystis* excretes small amounts of glutamate even during nitrogen starvation (Figure 1), when intracellular glutamate levels decrease considerably [22]. This is a clear indication against excretion as a result of passive metabolic overflow and suggests the contribution of an active transport mechanism that is triggered under specific metabolic conditions.

Our results also suggest that, while metabolite excretion in *Synechocystis* is primarily triggered by blocking the glycogen sink, it can be further modulated by other changes in the carbon flow. Despite accumulating WT levels of glycogen under nitrogen starvation, Δ*glgA1* and Δ*glgA2* excreted small amounts of glutamate (Figure 1), while displaying considerably lower intracellular 2-OG, glutamate, and glutamine levels compared to the WT (Figure 2). This suggests that deletion of either homolog subtly alters carbon flux partitioning and slightly increases residual GS/GOGAT activity compared to the WT, diverting more 2-OG into glutamate synthesis and excretion. Such reallocation of carbon flow could also explain the significant reduction in energy charge compared to the WT during chlorosis observed in the *glgA2* mutant. To date, no regulatory feedback of GlgA activity influencing nitrogen assimilation within *Synechocystis* has been described. However, our data suggest a potential connection between the glycogen synthases and the GS/GOGAT reaction, with glycogen synthase activity potentially influencing nitrogen assimilation.

We also observed that increasing carbon flux toward the TCA cycle via increased PGAM reaction through deletion of *pirC* [22] favored excretion of succinate and 2-OG at the expense of pyruvate excretion, as Δ*pirC* exclusively excreted 2-OG and succinate (Figure 1). This effect of *pirC* deletion could also be observed for the Δ*glgC*Δ*pirC* strain. Concomitant deletion of *pirC* and *glgC* only subtly altered metabolite excretion, with the double mutant excreting slightly more 2-OG and succinate than the Δ*glgC* single mutant. This was unexpected, as previous work had shown that Δ*pirC* accumulates substantially more pyruvate than succinate intracellularly after 48 h of nitrogen starvation [22]. However, we did not detect any extracellular pyruvate in the Δ*pirC* strain after two days of nitrogen depletion. Thus, Δ*pirC* did not excrete all metabolites that accumulated intracellularly, suggesting that metabolites are probably not simply excreted by passive diffusion but via a selective transport mechanism. This agrees with the work of Benson et al. [36], suggesting that an unknown transporter facilitates overflow excretion of pyruvate in *Synechococcus* PCC7942 and the hypothesis that overflow excretion is facilitated by mechanosensitive channels in glycogen-deficient cyanobacteria [31].

We also observed that strains accumulating metabolites to similar intracellular levels varied in the extent of metabolite excretion. The amount of 2-OG excreted by Δ*pgm* and especially Δ*pirC* was comparably small, despite Δ*pirC*, Δ*pgm*, Δ*glgC*, and Δ*glgC*Δ*pirC* displaying equally increased intracellular 2-OG levels upon nitrogen starvation. Similarly, while Δ*pirC* and Δ*glgC*Δ*pirC* had comparable intracellular succinate levels during nitrogen depletion, Δ*glgC*Δ*pirC* excreted considerably more succinate. Malate followed the same pattern, being excreted exclusively by Δ*pgm*, Δ*glgC*, and Δ*glgC*Δ*pirC*, even though intracellular malate levels were comparable across strains (Figure 1). Together, these findings suggest that excretion is not a simple function of intracellular metabolite abundance but may require metabolite levels to surpass a strain-specific threshold before being released.

This hypothesis is also supported by our observations concerning TCA cycle intermediates. Both WT predominantly accumulated downstream TCA intermediates such as succinate and malate upon nitrogen starvation. In accordance with the work of Kato et al. [31], disturbance of the glycogen sink within the metabolite-excreting mutants Δ*pirC*, Δ*pgm*, Δ*glgC*, and Δ*glgC*Δ*pirC* resulted in intracellular accumulation of TCA cycle intermediates during nitrogen starvation. Interestingly, this accumulation within the mutants was more pronounced within citrate/isocitrate and 2-OG, leading to stronger differences in early TCA cycle intermediate levels between the mutants and their respective WT. Simultaneously, only slight differences in succinate and almost no differences in the change of the malate levels could be observed (Figure 2). These observations could be explained by a threshold-dependent release of TCA intermediates, specifically 2-OG. The reaction generating 2-OG is a well-described bottleneck of the TCA cycle during nitrogen starvation. During nitrogen starvation, the GS/GOGAT comes to a halt, and 2-OG is no longer consumed for nitrogen assimilation, resulting in its buildup [24]. Threshold-dependent excretion of this accumulated 2-OG could provide a means of buffering the TCA cycle, thereby helping to maintain balanced levels of downstream TCA intermediates. Thus, downstream TCA intermediates only accumulate more pronouncedly than in the WT once overflow excretion is not sufficient to buffer increased carbon flow into the TCA. This was mainly observed in strains in which the carbon flow towards lower glycolysis and the TCA cycle was increased due to the deletion of *pirC*.

Remarkably, if metabolite excretion is triggered in a threshold-dependent manner, this threshold apparently lies above normal WT metabolite levels, as excretion did not consistently restore intracellular concentrations to WT values (Figure 1).

Metabolite excretion also did not result in global balancing of the intracellular metabolite levels. We observed stronger metabolic differences between strains excreting large amounts of metabolites and their respective WT than between strains with little or no excretion (Figure S2). Across most of the 124 measured intracellular metabolites, strains that excrete substantial amounts of metabolites (Δ*pgm*, Δ*glgC*, Δ*pirC*, and Δ*glgC*Δ*pirC*) showed a more pronounced increase in intracellular metabolites during nitrogen starvation compared to their respective WT. This was not observed for strains with minimal or no excretion (Figure S2). This indicates that while metabolic overflow is the result of a severely unbalanced metabolism, it is apparently not sufficient to restore WT metabolite balance. Nonetheless, excretion may act as a regulatory mechanism to prevent excessive accumulation and maintain metabolite levels within a defined physiological range.

Comparing the amount of carbon excreted as metabolites with that stored as glycogen revealed how excess carbon, which cannot be stored as glycogen, is redirected into the excretion of metabolites such as succinate, malate, pyruvate, and 2-OG (Figure 4). These findings highlight that metabolic overflow is important for intracellular homeostasis, as excess carbon and metabolites appear to be excreted using the medium as an alternative sink. Although overflow excretion alone is insufficient to maintain balanced metabolite levels, it might keep intracellular metabolite concentrations within a specific range that allows key regulatory and signaling processes to function. Previous publications indicate that metabolite-level regulation governs key processes. Doello et al. [29] described that the recovery from chlorosis and the enzymes required to return to vegetative growth are regulated by metabolite levels. Additionally, Carrieri et al. [37] suggested that overflow excretion is regulated at the metabolite level, by a difference in metabolic status, not a difference in sensing and responding to stress. Maintaining particularly hub metabolites, such as pyruvate and 2-OG, below critical levels may help cells maintain metabolic stability. 2-OG occupies a central role at the intersection of carbon and nitrogen metabolism and serves as an important regulatory signal [24,38], while pyruvate links glycolysis, fermentation, and amino acid synthesis [39,40], underscoring its central role in coordinating multiple metabolic pathways. Thus, accumulation of these hub metabolites exceeding specific critical levels may initiate excretion, acting as a buffer to protect central metabolism and enabling cells to respond efficiently to and during nitrogen starvation.

Another metabolite potentially influencing carbon partitioning and metabolic overflow is DHAP. We observed a correlation between high intracellular DHAP levels and metabolite excretion during nitrogen starvation for Δ*pgm*, Δ*glgC*, Δ*pirC*, and Δ*glgC*Δ*pirC* (Figure 2). DHAP is the precursor of methylglyoxal (MG), a toxic metabolic byproduct that is detoxified via conjugation of MG with reduced glutathione [41]. If DHAP accumulation in turn resulted in increased MG synthesis, it could trigger stress responses, such as rerouting the carbon flow into alternative carbon sinks, resulting in overflow excretion. However, we did not observe any indicative differences within the reduced glutathione pools of Δ*pgm*, Δ*glgC*, Δ*pirC*, and Δ*glgC*Δ*pirC* compared to their respective WT, which would suggest increased detoxification within these strains during nitrogen starvation (Figure 3C,F). Alternatively, changes in DHAP levels could also be part of transient changes within sugar-phosphate intermediates, which have been observed in *Synechocystis* during early chlorosis and are likely due to a shift in carbon partitioning [42,43]. As Δ*pgm*, Δ*glgC*, Δ*pirC*, and Δ*glgC*Δ*pirC* have a modified carbon flux due to their respective gene deletions, these changes in carbon partitioning might be simply more pronounced in these strains. This agrees with our results, which show an increase in intracellular hexose-P and pentose-P within all strains, with this increase being most pronounced in strains lacking *glgC* and therefore their glycogen sink.

Previous publications concluded that the intracellular redox and energy balance of *Synechocystis* plays an important role in the excretion of organic acids [21,32]. Here, we did not observe strong differences in redox or energy homeostasis during nitrogen starvation across strains, with the notable exception of the Δ*pgm* strain. However, our results suggest that the differences we observed within the adenylate pool as well as the NAD(H) and NADP(H) pools were not caused by differences within the redox or energy balancing of the various strains. Alternatively, the observed changes could be connected to changes in the purine metabolism. In *Synechocystis*, purine metabolism intermediates are substrates for nicotinamide nucleotide synthesis [33,44]. Interestingly, it has been shown that these intermediates additionally act as regulators of NAD synthesis in other organisms [45]. Thus, changes in purine metabolism could impact NAD(H) and NADP(H) dynamics in *Synechocystis*. We observed that purine metabolism is strongly downregulated in WT cells under nitrogen starvation, as seen by decreases in intermediates such as 5-amino-1-(5-phospho-D-ribosyl)imidazole and 5-amino-1-(5-phospho-D-ribosyl)imidazole-4-carboxamide (AICAR), with similar trends in the Δ*glnB* and Δ*glgA* strains (Figure S5). In contrast, Δ*pgm*, Δ*pirC*, Δ*glgC*, and Δ*glgC*Δ*pirC* mutants maintained considerably higher levels of downstream intermediates, including 5-carboxyamino-1-(5-phospho-D-ribosyl)imidazole, AICAR, adenylosuccinate, and, particularly in Δ*pgm*, elevated ADP and AMP levels. These differences in purine nucleotide pools could provide the basis for the altered NAD(H) and NADP(H) dynamics observed in the respective mutants. The observation that Δ*pgm* redirected fewer glucose equivalents into overflow excretion than the other glycogen-deficient mutants would also fit this theory. Instead of reallocating carbon flow into overflow excretion, Δ*pgm* might partially divert carbon flow into purine metabolism, resulting in greater differences within the Δ*pgm* adenylate, NAD(H), and NADP(H) pools. Additionally, the increased AMP accumulation accounts for the significantly reduced energy charge evident in Δ*pgm* during nitrogen depletion.

Interestingly, we also observed some differences between the glucose-sensitive and glucose-tolerant WT. During nitrogen chlorosis, the WT-GT did not decrease its intracellular glutamine and glutamate levels as strongly as the WT-GS (Figure 2). Additionally, despite differences in intracellular glutamine and glutamate levels, glutamate excretion was observed only in one WT-GS replicate. The WT-GT consistently showed no glutamate excretion, suggesting no reproducible differences in glutamate excretion between the two WT strains. Instead, small extracellular amounts of malate, succinate, and 2-OG were detected across all replicates of the nitrogen-starved WT-GS (Figure 1). These results suggest minor differences in carbon partitioning between glucose-sensitive and glucose-tolerant strains, particularly within the GS-GOGAT pathway. Furthermore, the overflow excretion of metabolites by WT-GS suggests that *Synechocystis* not only releases metabolites due to artificial changes in its carbon flux but also naturally excretes small amounts of specific metabolites during nitrogen starvation. It has been demonstrated that cyanobacteria growing in soil crusts release organic carbon to establish a resource trading relationship with heterotrophic nitrogen-fixing bacteria, exchanging carbon for combined nitrogen sources [46]. Furthermore, cyanobacteria within these communities utilize the excreted metabolites glutamate and mainly GABA as an interspecies signal for spatial organization [47]. Thus, excretion of specific metabolites by the WT might be part of the normal nitrogen starvation response as a mechanism to communicate with their environment or to attract other beneficial bacteria to help them cope with this shortage of nutrients. Further research is required to elucidate whether the excretion of specific metabolites by *Synechocystis* plays a role as an environmental signal.

## 5. Conclusions

Our findings reaffirm that blocking the glycogen sink in *Synechocystis* results in substantial carbon loss through the excretion of various metabolites, predominantly pyruvate and 2-OG. Beyond this, our results suggest that overflow excretion functions as an emergency mechanism ensuring that certain intracellular metabolite levels remain within a specific range.

This mechanism appears to be triggered by intracellular hub metabolite concentrations exceeding metabolite and strain-specific thresholds and is likely mediated by specific and active transport processes rather than passive diffusion. Since metabolite excretion does not restore WT metabolite balance, excretion thresholds probably exceed the WT metabolite levels. Furthermore, excretion of glutamate appears to result from metabolite-dependent activation of specific transport mechanisms. Together, these insights refine our understanding of carbon partitioning and overflow metabolism in *Synechocystis* and may inform strategies for future metabolic engineering.

## Figures and Tables

**Table 1:** ATP, ADP, and AMP C12/C13 ratios obtained from intracellular metabolite quantification via LC-MS/MS normalized to the OD_750_ of the respective cultures at the time of sampling (Table S2). The values represent intracellular metabolite levels of all strains after two days of vegetative growth (+N, green header) and after two days of nitrogen-depleted growth (−N, orange header). Each value represents the mean of a triplicate, including the SD.

	ATP		ADP		AMP	
	+N	−N	+N	−N	+N	−N
WT-GS	0.31 ± 0.15	0.06 ± 0.03	3.53 ± 1.68	1.84 ± 0.71	1.37 ± 0.39	1.15 ± 0.28
WT-GT	0.39 ± 0.10	0.15 ± 0.07	3.44 ± 0.90	1.18 ± 0.94	0.74 ± 0.06	0.50 ± 0.29
Δ*pgm*-GT	0.44 ± 0.19	0.38 ± 0.05	4.09 ± 0.73	5.63 ± 0.94	1.24 ± 0.08	7.35 ± 0.60
Δ*pirC*-GT	0.45 ± 0.21	0.51 ± 0.20	3.66 ± 1.53	2.77 ± 1.12	1.03 ± 0.18	0.78 ± 0.08
Δ*glgC*-GS	0.47 ± 0.12	0.13 ± 0.10	3.81 ± 0.65	2.66 ± 1.18	0.60 ± 0.28	2.15 ± 0.88
Δ*glgC*Δ*pirC*-GS	0.25 ± 0.02	0.28 ± 0.07	3.29 ± 1.66	6.14 ± 2.15	1.10 ± 0.64	3.32 ± 0.50
Δ*glnB*-GS	0.99 ± 0.12	0.14 ± 0.02	6.04 ± 1.15	4.58 ± 0.53	0.88 ± 0.24	2.90 ± 0.22
Δ*glgA1*-GT	0.29 ± 0.21	0.16 ± 0.07	3.18 ± 1.22	4.72 ± 1.71	1.14 ± 0.42	2.92 ± 0.85
Δ*glgA2*-GT	0.19 ± 0.05	0.05 ± 0.02	2.18 ± 0.21	1.59 ± 0.95	0.77 ± 0.29	2.60 ± 0.65

## Data Availability

After publication, the original data presented in this study will be openly available on FAIRDOMHub at https://fairdomhub.org/projects/510 (accessed on 28 November 2025). Further enquiries can be directed to the corresponding author.

## Supplementary Materials

The following supporting information can be downloaded at: https://www.mdpi.com/article/10.3390/microorganisms13122767/s1, Table S1: List of all strains used within this study; Table S2: Optical Density of all cultures at the time of sampling; Figure S1: Heat map depicting log2-fold changes in metabolites after 2 days of vegetative growth; Figure S2: Heat map depicting log2-fold changes in metabolites after 2 days of nitrogen starvation; Figure S3: Energy charge calculated from OD normalized ATP, ADP, and AMP C12/C13 ratios; Figure S4: Intracellular NAD/NADH and NADP/NADPH ratios; Figure S5: Purine metabolism; Equation (S1): Conversion of measured glycogen quantities into glucose equivalents per cell; Equation (S2): Conversion of measured amounts of excreted metabolites into intracellular metabolite concentrations removed from each cell via excretion. References [48,49,50] are cited in the Supplementary Materials.

## Abbreviations

The following abbreviations are used in this manuscript:

2-OG	2-oxoglutarate
AICAR	5-amino-1-(5-phospho-D-ribosyl)imidazole-4-carboxamide
AMG	amyloglucosidase
DHAP	dihydroxyacetone-phosphate
GlgA	glycogen synthase
GlgC	glucose-1-phosphate adenylyltransferase
GO	glucose oxidase
GS	glucose-sensitive
GS/GOGAT	glutamine synthetase/glutamate synthase cycle
GT	glucose-tolerant
HPLC-MS	high-performance liquid chromatography-mass spectrometry
LC-MS/MS	liquid chromatography-tandem mass spectrometry
MG	methylglyoxal
OD	optical density
ODD	*o*-dianisidine dihydrochloride
PEP	phosphoenolpyruvate
PGAM	phosphoglycerate mutase
PGM	phosphoglucomutase
PHB	polyhydroxybutyrate
PirC	P_II_-interacting regulator of carbon metabolism
PX	peroxidase
SD	standard deviation
*Synechocystis*	*Synechocystis* sp. PCC 6803
TCA	tricarboxylic acid
WT	wild type
WT-GS	glucose-sensitive wild type
WT-GT	glucose-tolerant wild type
