# Supplementary material for: Controlled Carbon Loss: Threshold-Dependent Overflow Metabolism in Synechocystis sp. PCC 6803
Source: Microorganisms. 2025 Dec 4;13(12):2767. doi: 10.3390/microorganisms13122767 (PMC12735552; doi:10.3390/microorganisms13122767)
Supplement: Supplementary file 1 [file microorganisms-13-02767-s001.zip › microorganisms-4004258-supplementary.pdf]

Supplemental Material

Table S1: List of all strains used within this study.

Strain	Background	Relevant Marker of Genotype	Reference
Synechocystis sp. PCC 6803 GS	glucose-sensitive (GS)	-	Pasteur culture collection
Synechocystis sp. PCC 6803 GT	glucose-tolerant (GT)	-	Chen et al. 2016 [48]
Δ*pgm*	glucose-tolerant (GT)	*sll0726*::specR	Doello et al. 2022 [16]
Δ*pirC*	glucose-tolerant (GT)	*sll0944*::specR	Orthwein et al. 2025 [24]
Δ*glgC*	glucose-sensitive (GS)	*slr1176*::cmR	This publication. Constructed by deleting *glgC* in the WT-GS background using pGLGC::Cm^R [49].
Δ*glgC*Δ*pirC*	glucose-sensitive (GS)	*slr1176*::cmR *sll0944*::kmR	This publication. Constructed by deleting *pirC* in the Δ*glgC* background using pJA1 [24].
Δ*glnB*	glucose-sensitive (GS)	*ssl0707*::specR	Hisbergues et al. 1999 [50]
Δ*glgA1*	glucose-tolerant (GT)	*sll0945*::kmR	Gründel et al. 2012 [17]
Δ*glgA2*	glucose-tolerant (GT)	*sll1393*::cmR	Gründel et al. 2012 [17]

Table S2: Optical Density at 750 nm (OD_{750}) of all cultures at the time of sampling for intracellular and extracellular metabolite quantification after two days of vegetative or nitrogen-depleted growth.

Culture	Optical Density at 750 nm (OD_{750})	
	**2 days +N**	**2 days -N**
WT-GT-1	1.46	1.08
WT-GT-2	1.42	1.05
WT-GT-3	1.45	1.02
WT-GS-1	1.16	1.06
WT-GS-2	1.33	0.97
WT-GS-3	1.35	1.04
Δ*pirC*-GT-1	0.98	0.87
Δ*pirC*-GT-2	0.75	0.75
Δ*pirC*-GT-3	1.28	0.74
Δ*pgm*-GT-1	0.9	0.42
Δ*pgm*-GT-2	0.96	0.42
Δ*pgm*-GT-3	1.01	0.42
Δ*glgA1*-GT-1	1.23	1.09
Δ*glgA1*-GT-2	0.88	1.09
Δ*glgA1*-GT-3	1.19	1.08
Δ*glgA2*-GT-1	0.82	1.12
Δ*glgA2*-GT-2	1.11	1.07
Δ*glgA2*-GT-3	1.22	1.16
Δp_{II}-GS-1	0.83	0.98
Δp_{II}-GS-2	1.41	0.91
Δp_{II}-GS-3	0.91	1.03
Δ*glgC*-GS-1	0.79	0.46
Δ*glgC*-GS-2	1.47	0.46
Δ*glgC*-GS-3	1.2	0.47
Δ*glgC*Δ*pirC*-GS-1	0.84	0.45
Δ*glgC*Δ*pirC*-GS-2	0.99	0.43
Δ*glgC*Δ*pirC*-GS-3	1.01	0.44

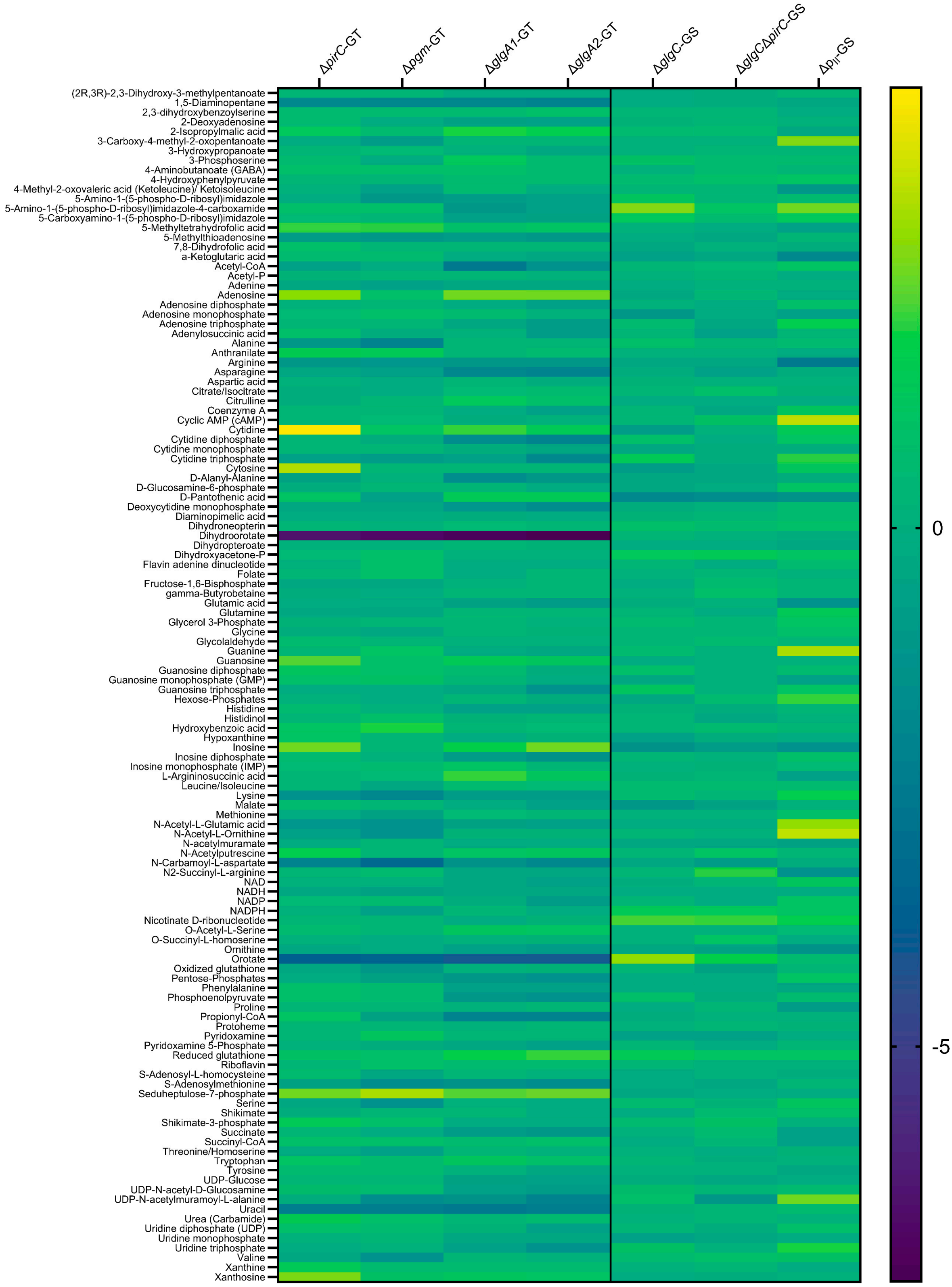

Figure S1. Heat map depicting log_2-fold changes in metabolites after 2 days of vegetative growth for WT-GT, Δ*pgm*-GT, Δ*pirC*-GT, Δ*glgA1*-GT, Δ*glgA2*-GT, WT-GS, Δ*glgC*-GS, Δ*glgC*Δ*pirC*-GS, and Δ*glnB*-GS, normalized to the metabolite levels of the corresponding WT during vegetative growth.

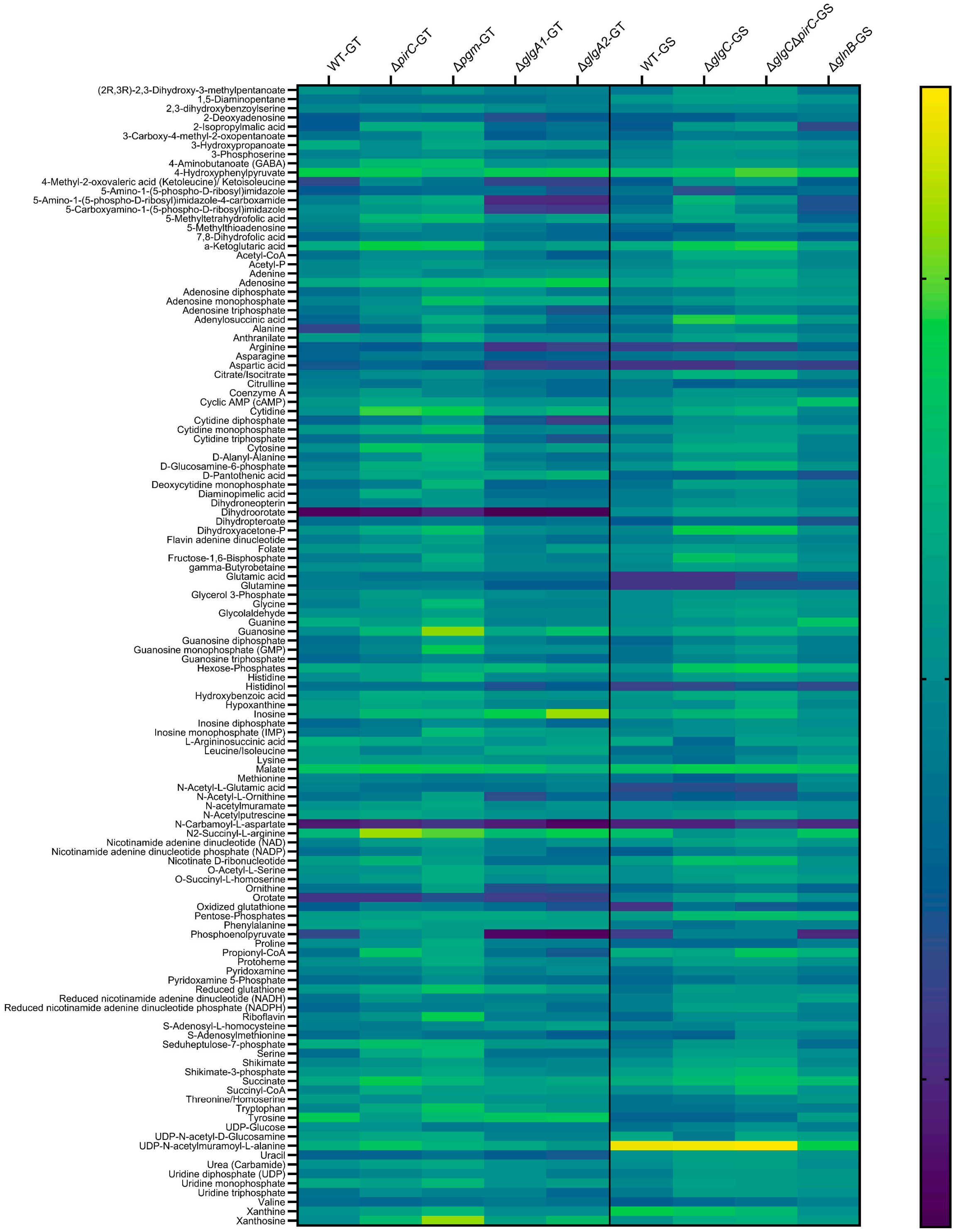

Figure S2. Heat map depicting log$_2$-fold changes in metabolites for WT-GT, Δ*pgm*-GT, Δ*pirC*-GT, Δ*glgA1*-GT, Δ*glgA2*-GT, WT-GS, Δ*glgC*-GS, Δ*glgC*Δ*pirC*-GS, and Δ*glnB*-GS after 2 days of nitrogen starvation, normalized to the metabolite levels of the corresponding WT during vegetative growth.

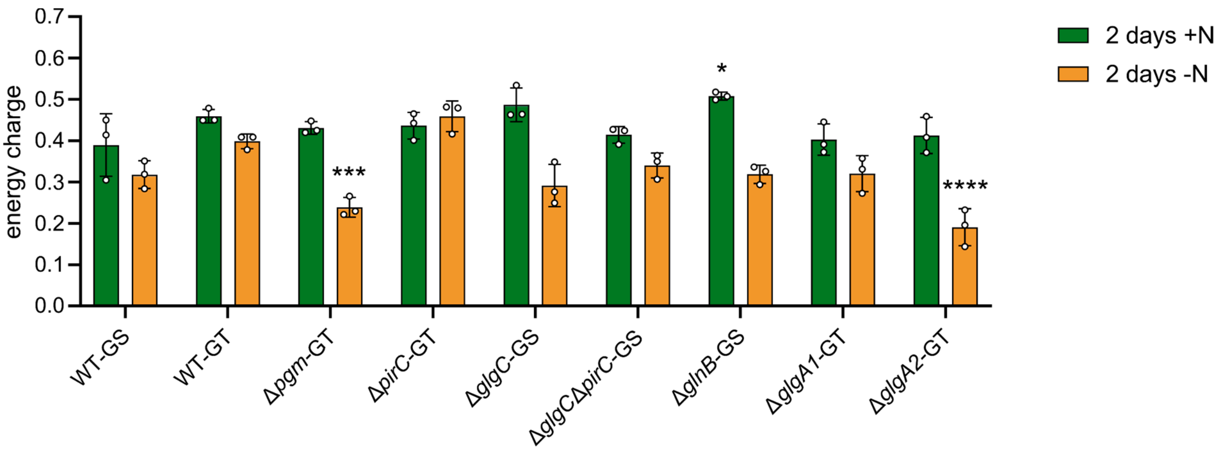

Figure S3. Energy charge calculated from OD normalized ATP, ADP, and AMP C12/C13 ratios of WT-GS, WT-GT, Δ*pgm*-GT, Δ*pirC*-GT, Δ*glgC*-GS, Δ*glgC*Δ*pirC*-GS, Δ*glnB*-GS, Δ*glgA1*-GT, and Δ*glgA2*-GT after two days vegetative growth (green) and after 2 days of nitrogen depletion (orange). The bars represent the mean values of triplicates, including the SD, and the individual values are depicted as dots. The values of all strains were compared to those of their respective WT cultivated under the same conditions by performing an ordinary one-way ANOVA. Asterisks (*) in the figures symbolize p values: one asterisk corresponds to $p \leq 0.05$, three asterisks to $p \leq 0.001$, and four asterisks to $p \leq 0.0001$.

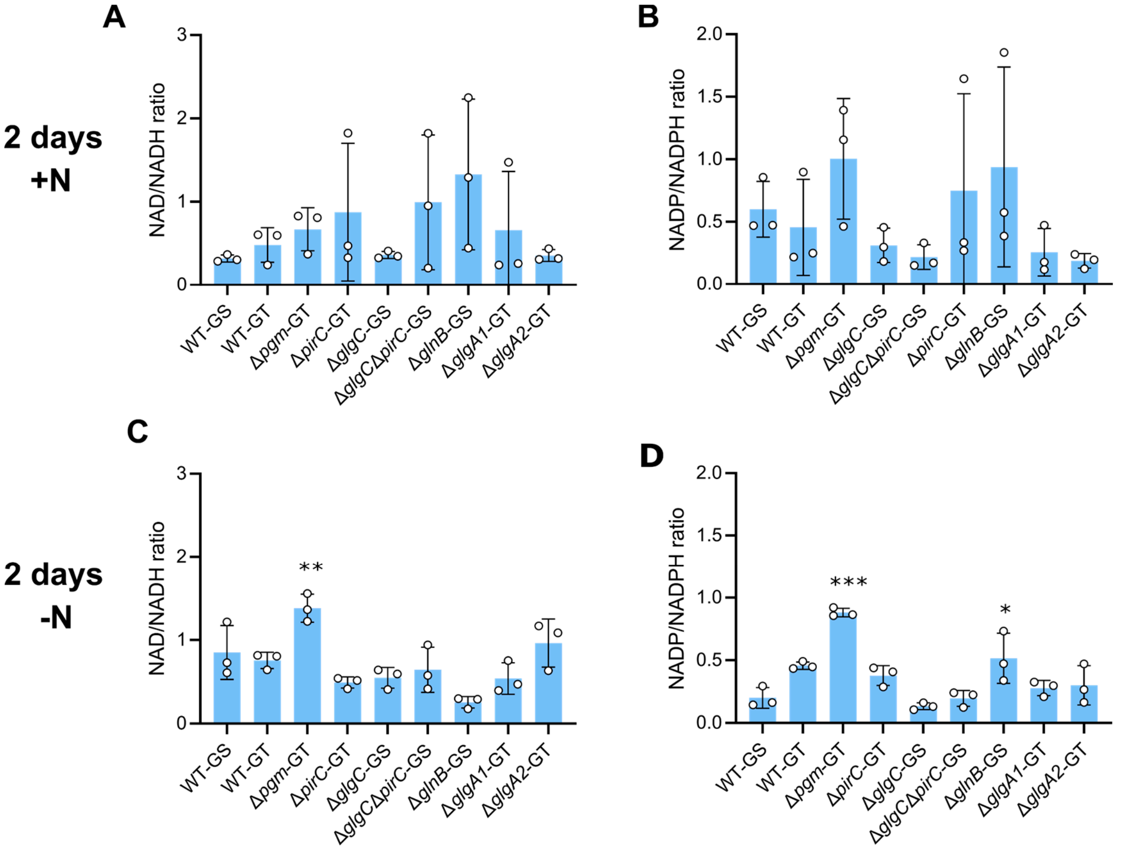

Figure S4. Intracellular NAD/NADH (A and C) and NADP/NADPH (B and D) ratios of WT-GS, WT-GT, Δ*pgm*-GT, Δ*pirC*-GT, Δ*glgC*-GS, Δ*glgC*Δ*pirC*-GS, Δ*glnB*-GS, Δ*glgA1*-GT, and Δ*glgA2*-GT after two days of vegetative growth (A and B) and after 2 days of nitrogen depletion (C and D). Each bar represents the mean of a triplicate, including SD, and the individual values are depicted as dots. The values of all strains were compared to those of their respective WT cultivated under the same conditions by performing an ordinary one-way ANOVA. Asterisks (*) in the figures symbolize p values: one asterisk corresponds to $p \leq 0.05$, two asterisks to $p \leq 0.01$ and three asterisks to $p \leq 0.001$.

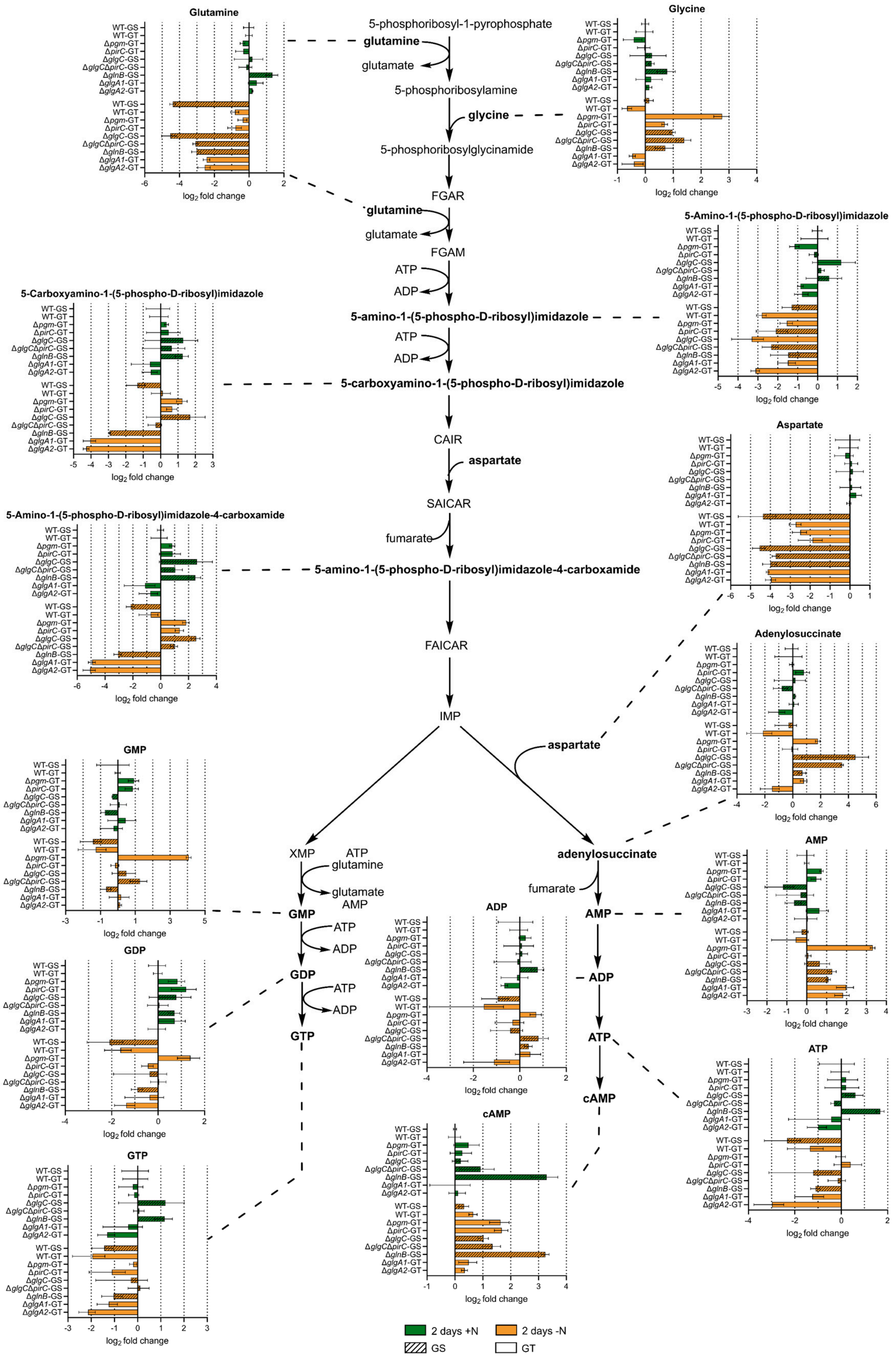

Figure S5. Purine metabolism. Log$_2$-fold changes in metabolites for WT-GS, WT-GT, Δ*pgm*-GT, Δ*pirC*-GT, Δ*glgC*-GS, Δ*glgC*Δ*pirC*-GS, Δ*glnB*-GS, Δ*glgA1*-GT, and Δ*glgA*2-GT after 2 days of vegetative growth (green) or 2 days of nitrogen starvation (orange), normalized to the metabolite levels of the

corresponding WT during vegetative growth. Each bar represents the mean $\log_2$-fold change of a triplicate, including the negative and positive SD.

$$glycogen\ [\frac{\mu g}{cell}] = \frac{glycogen\ [\frac{\mu g}{ml}]}{OD * 10^8 [\frac{cells}{ml}]}$$

$$glucose\ [\frac{\mu mol}{cell}] = \frac{glycogen\ per\ cell\ [\frac{\mu g}{cell}]}{180.156\ [\frac{\mu g}{\mu mol}]}$$

$$intracellular\ glucose\ [\frac{mol}{l}] = \frac{intracellular\ glucose\ per\ cell\ [\frac{\mu mol}{cell}] * 10^{-6}}{3.8\ * 10^{-15}\ [\frac{l}{cell}]}$$

$$intracellular\ glucose\ [mM] = intracellular\ glucose\ \left[\frac{mol}{l}\right] * 1000$$

Equation S1. Conversion of measured glycogen quantities into glucose equivalents per cell. The calculation was performed based on the assumptions that an OD_{750} of 1 corresponds to $1*10^8$ cells and that cells are spherical with a diameter of ~1.7–2.0 µm and an average volume of ~3.8 μm^3.

$$factor\ extra-/intracellular\ volume = \frac{V_{extracellular}\ [ml]}{N_{cells}\left[\frac{cells}{ml}\right] * V_{intracellular}\left[\frac{ml}{cell}\right] * V_{total}\ [ml]}$$

$$factor\ = \frac{1\ [ml] - V_{intracellular}}{\left(OD * 10^8 \left[\frac{cells}{ml}\right]\right) * \left(3.8\ * 10^{-12}\ \left[\frac{ml}{cell}\right]\right) * 1\ [ml]}$$

$$factor = \frac{1\ [ml]}{\left(OD * 10^8 \left[\frac{cells}{ml}\right]\right) * \left(3.8\ * 10^{-12}\ \left[\frac{ml}{cell}\right]\right) * 1\ [ml]}$$

$$c_{\text{intracellular}}\ \text{removed via excretion} = factor\ * extracellular\ metabolite\ concentration\ [mM]$$

Equation S2. Conversion of measured amounts of excreted metabolites into intracellular metabolite concentrations removed from each cell via excretion. The calculation was performed based on the assumptions that an OD_{750} of 1 corresponds to $1*10^8$ cells, that cells are spherical with a diameter of ~1.7–2.0 µm and an average volume of ~3.8 μm^3, and that $V_{intracellular}$ is negligibly small.
